# Modulatory Effects of *Bacillus subtilis* on the Performance, Morphology, Cecal Microbiota and Gut Barrier Function of Laying Hens

**DOI:** 10.3390/ani11061523

**Published:** 2021-05-24

**Authors:** Guangzhi Zhang, Hao Wang, Jianwei Zhang, Xinming Tang, Abdul Raheem, Mingyan Wang, Weidong Lin, Lin Liang, Yuzhuo Qi, Yali Zhu, Yaxiong Jia, Shangjin Cui, Tong Qin

**Affiliations:** 1 Institute of Animal Sciences, Chinese Academy of Agricultural Sciences, Beijing 100193, China; zhangguangzhi@caas.cn (G.Z.); wanghao_3001@163.com (H.W.); tangxinming@caas.cn (X.T.); 2019y90100011@caas.cn (A.R.); mingyan.wang@ceva.com (M.W.); linweidong@caas.cn (W.L.); lianglin@caas.cn (L.L.); qyz0314@163.com (Y.Q.); zyl41018@126.com (Y.Z.); 2 Scientific Observation and Experiment Station of Veterinary Drugs and Diagnostic Technology of Beijing, Ministry of Agriculture, Beijing 100193, China; 3 Beijing General Station of Animal Husbandry, Beijing 100107, China; zjw7432@126.com

**Keywords:** *Bacillus subtilis*, cecal microbiota, barrier function, occludin, probiotic

## Abstract

**Simple Summary:**

The excessive or improper use of antibiotics in chicken feed has led to the emergence of antibiotic-resistant bacteria, drug residues in the tissue, and other relevant issues, and this situation is severe in China. From 31 December 2020, antibiotics were banned for use as supplemental growth promoters in animal feed in China; therefore, antibiotic substitutes are urgently needed in China. People are searching for ideal antibiotic alternatives, and probiotics have proven their potential use for this. Our current trial aims to evaluate the effects of the *Bacillus subtilis* (*B. subtilis*) YW1 strain on laying hens, including the effects on performance, morphology, cecal microbiota, and intestinal barrier function. Our study showed that orally administering laying hens with probiotic *B. subtilis* cannot significantly improve their overall egg production, can induce a healthier microbiota composition characterized by a higher ratio of beneficial bacteria, and strengthens the physical barrier function of the intestine by inducing a higher expression of the tight junction protein. This makes it a valid probiotic alternative to antibiotics, and also a reference strain for clinical application in the poultry industry.

**Abstract:**

We investigated the efficacy of a single bacterium strain, *Bacillus subtilis* (*B. subtilis*) YW1, on the performance, morphology, cecal microbiota, and intestinal barrier function of laying hens. A total of 216 28-week-old Hy-line Brown laying hens were divided into three dietary treatment groups, with six replicates of 12 birds each for 4 weeks. The control group (Ctr) was fed a basal diet and the treatment groups, T1 and T2, were fed a basal diet supplemented with *B. subtilis* at a dose rate of 5 × 10^8^ CFU/kg and 2.5 × 10^9^ CFU/kg, respectively. Dietary supplementation with *B. subtilis* did not significantly affect overall egg production in both groups, with no obvious changes in average egg weight and intestine morphology. *B. subtilis* administration also improved the physical barrier function of the intestine by inducing significantly greater expression levels of the tight junction protein occludin in T1 (*p* = 0.07) and T2 (*p* < 0.05). Further, supplementation with *B. subtilis* effectively modulated the cecal microbiota, increasing the relative level of beneficial bacteria at the genus level (e.g., *Bifidobacterium* *p* < 0.05, *Lactobacillus p* = 0.298, *Bacillus* *p* = 0.550) and decreasing the level of potential pathogens (e.g., *Fusobacterium p* < 0.05, *Staphylococcus p* < 0.05, *Campylobacter p* = 0.298). Overall, *B. subtilis* YW1 supplementation cannot significantly improve the egg production; however, it modulated the cecal microbiota towards a healthier pattern and promoted the mRNA expression of the tight junction protein occludin in laying hens, making *B. subtilis* YW1 a good probiotic candidate for application in the poultry industry, and further expanding the resources of strains of animal probiotics.

## 1. Introduction

In order to inhibit the pathogenic bacteria in the gut and improve feed conversion ratio and meat production, chicken feed could be supplemented with a daily low dose of antibiotics, especially in commercial poultry farms worldwide [1,2]. However, increasing evidence indicates that the excessive or indiscriminate use of antibiotics in the feed of chickens and livestock may lead to the emergence of antibiotic-resistant bacteria or drug residues in the tissue, and may also compromise the efficacy of antibiotic treatment [3,4,5]. Further, this excessive use of antibiotics poses a threat to public health due to the potential transfer of antibiotic resistance genes between animal-derived products and humans. Thus far, antibiotic resistance (ABR) has been a worldwide issue in both farm animals and public health. Supplementing feed with antibiotics as a growth promoter is already prohibited in Europe, the U.S., and some other countries, such as China, which banned antibiotics supplementation in feed in 2020 [6]. Paradoxically, the prohibition of antibiotics in livestock and poultry feed has caused a relative increase in bacterial infection, and reduced the quality and performance of animals and poultry [2,7]. As such, researchers are searching for ideal antibiotic substitutes, and numerous studies have shown that some probiotics and prebiotics possess health-promoting and disease-preventing abilities in both humans and animals.

Probiotics are defined as “live microorganisms which when administered in adequate amounts confer a health benefit on the host” by the World Health Organization [8]. Considerable efforts are being made to find probiotic alternatives to antibiotics and, currently, the majority of probiotics employed in chicken farming are *Lactobacillus* (*L.*) spp., *Bifidobacterium* spp., *Bacillus* (*B.*) spp., or *Enterococcus* spp. [6,9,10,11]. Tailored probiotics can modulate the microbiome, mycobiome, and host gene expression of turkeys in a manner similar to low doses of antibiotic growth promoter [12]. The administration of *L. salivarius* or *L. plantarum* in feed significantly increases the levels of short-chain fatty acids and induces a healthier pattern in the intestinal microbiota of broiler chickens [13,14]. Dietary supplementation with *B. subtilis* has positive effects on productivity, the stimulation of immune activities, and the improvement of antioxidant capacity in broilers [15]. Dietary supplementation with *Enterococcus faecalis* strain UGRA10 increases the ileum and caecum bacterial diversity in laying hens [16]. Amongst the probiotic species, *Bacillus* are popular feed additives due to their spore-producing abilities, which can counteract the extreme conditions and stresses the host is subjected to.

We have evaluated the safety and potential effects of *B. subtilis* on the performance, morphology, gut barrier function, and cecal microbiota of laying hens after administration for 4 weeks.

## 2. Materials and Methods

### 2.1. Probiotic Preparation

The *Bacillus subtilis* strain YW1 used in this study was isolated, characterized, and stored by our lab; this strain showed a high acid-resistant ability and antimicrobial ability in vitro. *B. subtilis* was added to basal feed as a freeze-dried powder. The freeze-dried *B. subtilis* was supplemented homogeneously to the basal diet up to 5 × 10^8^ CFU/kg and 2.5 × 10^9^ CFU/kg.

### 2.2. Laying Hens and Farm Facilities

The experimental group consisted of 216 28-week-old Hy-line Brown laying hens, and was divided into 3 groups, each containing 6 replicates with 12 birds in 1 replicate. The control group (Ctr) received only the basal diet, treatment group 1 (T1) received the basal diet supplemented with *B. subtilis* at a dosage of 5 × 10^8^ CFU/kg, and treatment group 2 (T2) received the basal diet supplemented with *B. subtilis* at a dosage of 2.5 × 10^9^ CFU/kg for 4 weeks. In this experiment, the chickens were fed a corn–soybean basal diet, which was antibiotic-free and formulated to meet the nutrient requirements of laying hens.

The ingredients of the basal diet used in this study are shown in Table 1.

Cage distribution was equal between the upper and lower cage levels to minimize any effect of the cage level. All the chickens were housed in steel wire cages (57 × 47 × 47 cm) with 3 birds per cage. The ambient temperature and room humidity of the farm facility were maintained at 19 ± 2 °C and 45~65%, respectively. The photoperiod was set to 16:8 light:dark throughout the study.

## 3. Experimental Procedure

### 3.1. Ethics Statement

This bird trial was evaluated and approved by the local ethic committee of the Institute of Animal Science, Chinese Academy of Agricultural Science (IAS2019-71), China. The experimental protocol was carried out in strict accordance with the relevant guidelines.

### 3.2. Hens Performance

Eggs from each replicate were collected and counted once a day. Egg weight was monitored daily and was calculated as the mean weight of all eggs from each replicate.

### 3.3. Intestinal Morphology

At the end of the trial, one bird from each replicate was randomly selected for euthanasia. Approximately 5 cm of the jejunum was cut from the midpoint between the point of bile duct entry and Meckel’s diverticulum. The tissue was gently flushed several times with physiological saline (1% NaCl) to remove intestinal contents, and then placed in 10% formalin for fixation. The intestinal samples were dehydrated and embedded in paraffin following routine procedures. A 3 μm thick tissue section was taken and stained with hematoxylin and eosin for histological observation. The intestinal morphology was evaluated via light microscopy (Olympus, Tokyo, Japan).

The villus’ height and crypt depth were measured in around five to seven randomly selected villi and associated crypts per hen, at 10× combined magnification, and the villus height/crypt depth ratio (VCR) was also calculated.

### 3.4. Quantitative Real-Time PCR

To determine the immune-related gene transcript levels in the jejunum, total RNA was extracted from the jejunal tissue with Trizol reagent (Ambion, Carishad, CA, USA), according to the manufacturer’s protocol as described elsewhere [17]. In brief, jejunal tissue (~100 mg) was collected and kept in 1 mL of RNAlater solution (Invitrogen, Vilnius, Lithuana) at −70 °C until further RNA extraction. The tissue was first homogenized with IKA T10 basic ULTRA-TURRAX (IKA, Königswinter, Germany), and then resuspended with Trizol reagent, followed by phase separation via chloroform and RNA precipitation and, finally, clean-up. The RNA concentration and purity were measured using NanoVue Plus (Biochrom, Cambridge, UK).

The RNA concentration of all samples was adjusted to 0.5 μg/μL, and cDNA was synthesized immediately from 2 μg of purified RNA using the FastKing RT Kit with gDNase (Tiangen Biotech Co., Ltd., Beijing, China).

The housekeeping genes β-Actin and GAPDH were used as reference genes. The mRNA expression levels of the target genes (*Occludin*, *TLR1*, *TLR2*, *TLR4*, *TLR15*, *TNFSF15*, *LYZ*) were quantified using SYBR Green-based RT-PCR with ChamQ Universal SYBR qPCR Master Mix (Vazyme Biotech Co., Ltd., Nanjing, China). The primers were synthesized with BGI (Beijing, China), and are shown in Table 2. The reactions were performed using a CFX96 RT-PCR System in a C1000 Thermal Cycler (Bio-Rad). All reactions were performed in 20 μL volumes containing 0.5 μL of each primer (1.25 pmol/μL), 10 μL ChamQ Universal SYBR qPCR Master Mix, 7 μL HPLC water, and 2 μL cDNA. The experimental program was as follows: 95 °C for 30 s, followed by 40 cycles of denaturation at 95 °C for 10 s, annealing at 60 °C for 30 s, and extension at 72 °C for 30 s. The melting curves were analyzed for the specific amplifications of all genes. The threshold cycle values (Ct) were normalized to the geometric means of the reference genes, and the normalized mRNA levels of all target genes were calculated using the 2^−ΔΔCt^ method [18].

### 3.5. Cecal Microbiota Analysis by 16S rRNA High-Throughput Sequencing

The cecal content was collected, snap-frozen, and kept at −70 °C until further DNA extraction. DNA was extracted using the E.Z.N.A. ^®^Stool DNA Kit (D4015, Omega, Inc., Norcross, GA, USA), according to the manufacturer’s instructions. A reagent designed to extract DNA from trace amounts of sample has been shown to be effective for the preparation of DNA from most bacteria. Nuclease-free water was used for the blank. The total DNA was eluted in 50 μL of elution buffer and stored at −80 °C until measurement in the PCR at LC-Bio Technology Co., Ltd., Hang Zhou, Zhejiang province, China.

The V3–V4 region of 16S rRNA gene was amplified with primers 341F (-5’CCTACGGGNGGCWGCAG3’ and 805R -5’GACTACHVGGGTATCTAATCC3’. The 5’ ends of the primers were tagged with specific barcodes per sample and sequencing universal primers. PCR amplification was performed in a 25 μL total volume of reaction mixture containing 25 ng of template DNA, 12.5 μL PCR premix, 2.5 μL of each primer, and PCR-grade water to adjust the volume. The PCR program for amplifying the prokaryotic 16S fragments was as follows: initial denaturation at 98 °C for 30 s; 32 cycles of denaturation at 98 °C for 10 s, annealing at 54 °C for 30 s, and extension at 72 °C for 45 s; and then final extension at 72 °C for 10 min. The PCR samples were checked with 2% agarose gel electrophoresis. Throughout the DNA extraction process, ultrapure water (instead of sample solution) was used as a negative control to prevent false-positive PCR results. The PCR samples were purified with AMPure XT beads (Beckman Coulter Genomics, Danvers, MA, USA) and quantified via Qubit (Invitrogen, Waltham, MA, USA). The amplicon pools were prepared for further sequencing. The sizes and quantities within the amplicon library were assessed using an Agilent 2100 Bioanalyzer (Agilent, Santa Clara, CA, USA) and the Library Quantification Kit for Illumina (Kapa Biosciences, Woburn, MA, USA), respectively. The libraries were sequenced on the Nova Seq PE250 platform.

### 3.6. Sequencing Data Analysis

The samples were sequenced on an Illumina NovaSeq platform from LC-Bio, based on the manufacturer’s recommendations. Paired-end reads were assigned to samples based on their unique barcode, and then truncated by cutting off the barcode and primer sequence. The paired-end reads were merged using FLASH. The raw tags were quality-filtered under specific filtering conditions to obtain high-quality clean tags according to the fqtrim (V 0.94). Chimeric sequences were filtered using Vsearch software (v2.3.4). Sequences with ≥97% similarity were assigned to the same operational taxonomic unit (OTU) by Vsearch (v2.3.4). Representative sequences were chosen for each OTU, and taxonomic data were then assigned to each representative sequence using the RDP (ribosomal database project) classifier. The OTU abundance information was normalized using a standard sequence number corresponding to the sample with the least sequences. Alpha diversity was used to analyze the species diversity of a sample via several indicators, such as Chao1, Shannon, Simpson, etc., all of which were calculated with QIIME (Version 1.8.0). Beta diversity analyses were used to assess differences in complexity between species, and were calculated via PCoA and cluster analysis with QIIME software. Blast was used for sequence alignment, and the OTU representative sequences were annotated with the RDP (ribosome database) and NCBI-16S database for individual representative sequences.

### 3.7. Statistical Analysis

The differences in performance and the mRNA expression levels of different genes in laying hens were assessed with one-way ANOVA, followed by a Bonferroni post hoc test. Differences in the composition and relative abundance of microbiota were assessed with the Wilcoxon rank-sum test. Each replicate served as the experimental unit for the parameters examined, and *p* values less than 0.05 were considered statistically significant. All data are expressed as mean ± SD. The clean data were analyzed and presented using Majorbio Cloud (Majorbio Bio-Pharm Technology Co., Ltd., Shanghai, China). The rest of the figures were created using GraphPad Prism5 software (GraphPad Software Inc., San Diego, CA, USA).

## 4. Results

### 4.1. Effects of B. subtilis Supplementation on Overall Performance and Morphology of the Jejunum of Laying Hens

No birds died or showed any signs of clinical disease throughout the experimental trial. Prior to *B. subtilis* supplementation, baseline egg production and egg weight were monitored for 1 week. No significant difference was observed between control group and T1, T2 groups in terms of egg production (Table 3) and egg weight (Table 3). As shown in Table 3, compared to the control group fed a normal diet, egg production in T1 and T2 (fed diets with *B. subtilis* supplementation) did not show a significant increase over the whole experimental period. Average egg weight showed no significant differences between any groups (Table 3, *p* > 0.05).

Similarly, compared to the control group, no statistically significant differences in villus height (*p* > 0.05), crypt depth (*p* > 0.05) or villus height/crypt depth ratio (*p* > 0.05) were observed in either treatment group (T1, T2) (Table 4).

### 4.2. Effects of B. subtilis Supplementation on Gut Microbiota of Laying Hens

**Figure 1:**
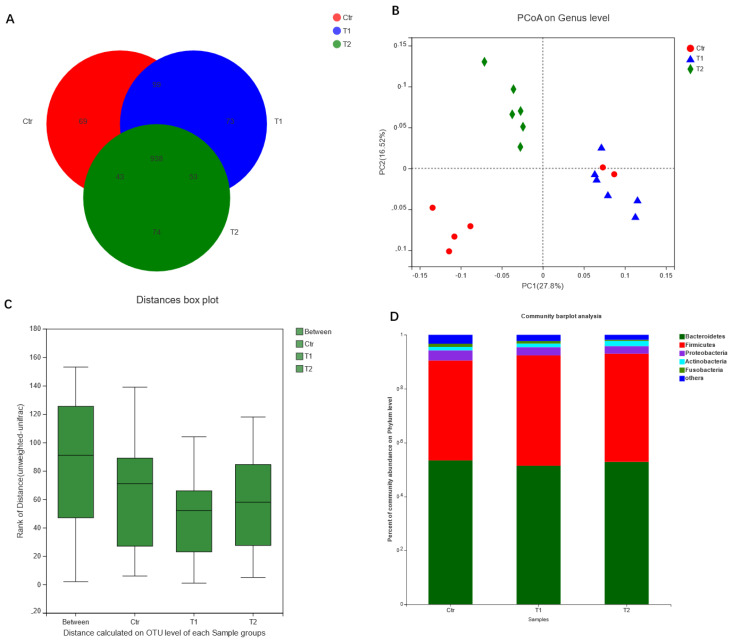
Effects of *B. subtilis* supplementation on the composition and abundance of cecal microbiota: (**A**) Venn diagram of OTUs in three groups. (**B**) Principal coordinate analysis (PCoA) of bacterial beta diversity based on the unweighted UniFrac distances in the three groups. The percentage of variation given by the plotted principal components is marked on both axes. Each spot represents one sample. (**C**) ANOSIM analysis based on unweighted UniFrac distances. (**D**) Composition of most dominant microbiota at the phylum level. Ctr: control group fed a basal diet (*n* = 6); T1: group fed a basal diet supplemented with *B. subtilis* (5 × 10^8^ CFU/kg) (*n* = 6); T2: group fed a basal diet supplemented with *B. subtilis* (2.5 × 10^9^ CFU/kg) (*n* = 6).

As shown in Figure 1A, T1 exhibited the greatest number of OUTs, and three groups shared 938 of the OUTs of cecal microbiota. The species richness parameters (Alpha diversity) exhibited no significant differences. PCoA analysis (Beta diversity) revealed a significant distinction between Ctr, T1 and T2 based on unweighted UniFrac distances (Figure 1B). Additionally, ANOSIM analysis, based on unweighted UniFrac distances, showed that the variation between groups was higher than the variation between samples within a single group (R = 0.370, *p* = 0.001, Figure 1C). The analysis of dominant microbiota at the phylum level showed a similar pattern among the three groups (Figure 1D).

**Figure 2:**
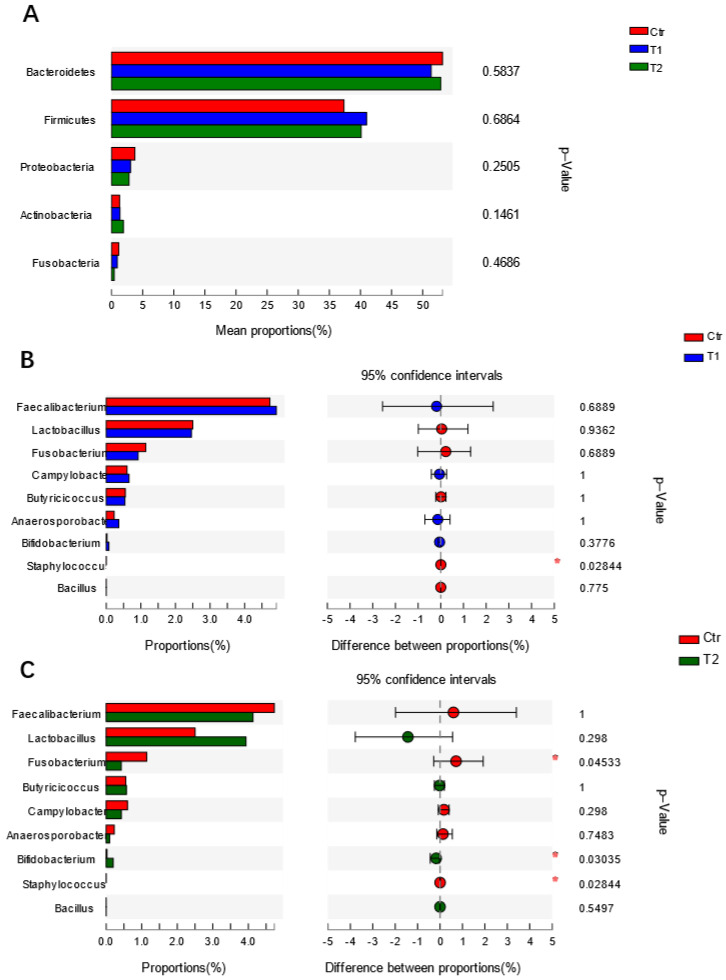
Effects of *B. subtilis* supplementation on the relative abundances of microbiota at the phylum and genus levels: (**A**) Top 5 most dominant phyla in chickens fed different diets. (**B**) Relative abundances of representative microbiota at the genus level between the control group and T1. (**C**) Relative abundances of representative microbiota at the genus level between the control group and T2. Statistical analysis was performed by the Wilcoxon rank-sum test. * indicates a statistically significant difference (*p* < 0.05) between the control group and the probiotic-treated groups (T1, T2). T1: group fed a basal diet supplemented with *B. subtilis* (5 × 10^8^ CFU/kg); T2: group fed a basal diet supplemented with *B. subtilis* (2.5 × 10^9^ CFU/kg).

As for the difference in microbiota composition, no significant alterations were observed in the top five most dominant phyla in chickens fed with different diets (Figure 2A). Further analysis of microbiota composition at the genus level showed significant variations in one group of genera (Figure 2A). Compared to Ctr, the relative abundances of 19 and 29 genera were significantly changed in T1 and T2, respectively (parts of the data are shown in Figure 2B,C). Compared to Ctr, the relative abundance of *Staphylococcus* (*p* < 0.05) was reduced (Figure 2B). Moreover, the relative abundances of specific potential pathogens in T2 were reduced or showed a reducing trend, e.g., *Fusobacterium* (*p* < 0.05), *Staphylococcus* (*p* < 0.05), *Campylobacter* (*p* = 0.298). Conversely, the relative abundances of certain beneficial bacteria in the T2 group increased or showed an increasing trend, e.g., *Bifidobacterium* (*p* < 0.05), *Lactobacillus* (*p* = 0.298), *Bacillus* (*p* = 0.550) (Figure 2C). However, the changes in relative abundance were not obvious (Figure 2B). Moreover, compared to Ctr, the relative abundances of other potential pathogens were also significantly reduced, e.g., Enterobacter, Vibrio (data not shown).

### 4.3. Changes in Gene mRNA Expression Level in Response to B. subtilis Administration

**Figure 3:**
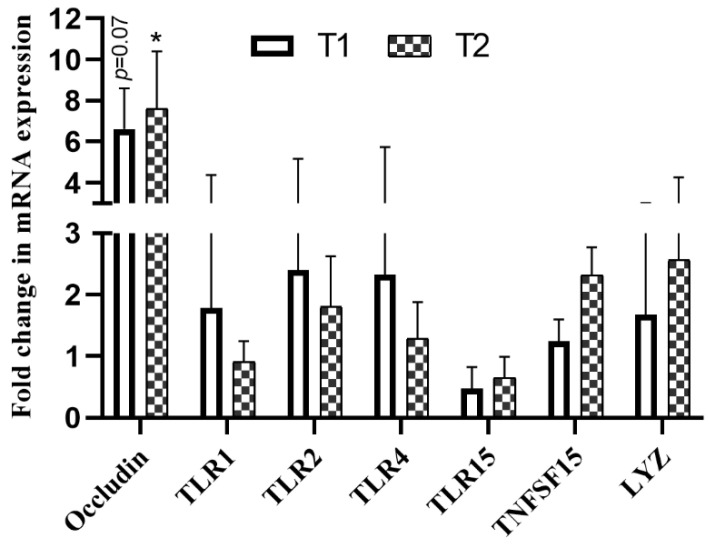
Effects of *Bacillus subtilis* on mRNA expression of tight junction protein and other immune-related genes. Mean changes in mRNA expression of several genes in chickens fed a basal diet or a diet supplemented with different doses of *B. subtilis*. The mean fold change in the control group is equal to 1. * indicates a statistically significant difference (*p* < 0.05) between the control and probiotic-treated groups. T1: group fed a basal diet supplemented with *B. subtilis* (5 × 10^8^ CFU/kg); T2: group fed a basal diet supplemented with *B. subtilis* (2.5 × 10^9^ CFU/kg).

As regards the tight junction protein occludin, T2, fed a diet supplemented with *B. subtilis* (2.5 × 10^9^ CFU/kg), displayed a statistically significant upregulation compared to chickens fed a basal diet (Figure 3). A similar expression trend was observed in T1; however, there was no statistically significant difference (*p* = 0.07). No significant changes in TLR1, TLR2, TLR4, TLR15, TNFSF15, or LYZ mRNA expression levels were observed between groups (Figure 3).

## 5. Discussion

*B. subtilis* supplementation did not significantly improve the average egg production of laying hens after feeding for 4 weeks, but it can induce a healthier microbiota composition and a higher expression of gut tight junction protein. The results prove that *B. subtilis* YW1 could serve as a potential probiotic replacement of antibiotics, and that further research on long-term *B. subtilis* YW1 administration is needed to confirm the effects.

Probiotics are deemed an ideal antibiotic alternative for poultry, livestock, and other farm animals, and a group of probiotic strains has been tested for their modulatory effects on animals’ growth, performance, gut physical barrier functions, immune response, intestinal ABR gene profiles, microbiota composition, etc. [13,24,25,26,27]. *L. plantarum* B1 administration can improve the performance of broilers, modulate their gut microbiota composition, and elevate their short-chain fatty acid (SCFA) levels [14]. *B. licheniformis* can alleviate heat stress-induced impairments of egg production [28]. Probiotic *B. subtilis* can increase laying performance via several mechanisms [29,30,31]. Besides *Bacillus* and *Lactobacillus*, many other bacteria are used in the poultry industry as probiotics, such as *Clostridium butyricum*, which can counteract the diverse stresses in the body and has the ability to improve laying hens’ performance and egg quality [32,33]. The present study showed that *B. subtilis* supplementation in the diets of laying hens can non-statistically significantly elevate overall egg production, especially in those supplemented with a low dose (5 × 10^8^ CFU/kg) of *B. subtilis*, without affecting the average egg weight. This could be because higher dosages of probiotics may consume more of the nutrients that birds use for egg production, but no data are available on this, so the underlying mechanisms require further exploration. Our results agree with those of many other studies, which also showed that the administration of *B. subtilis* DSM29784 for over 30 weeks does not significantly increase egg production. Similar results were also observed in *B. subtilis* CGMCC1.921- and *B. subtilis* PB6-treated hens [30,34,35]. *Enterococcus faecalis* UGRA10 can be supplemented into the diet to enhance egg production during the second half of the experimental period (40–76 days), but not during first half [16]. Our current study only lasted for 4 weeks, and so considering the duration of the production period of laying hens, further experiments employing long-term administration of *B. subtilis* are necessary.

Consistent with a previous study [36], *Bacteriodetes*, *Firmicutes*, *Proteobacteria*, *Actinobacteria*, and *Fusobacteria* are the dominant phyla in the cecum of laying hens. Normally, a higher bacterial richness index means a stable ecology to cope with the complex environment. In our study, no significant increase in the bacterial richness index (Alpha diversity) was shown in the group treated with *B. subtilis*. This reveals that *B. subtilis* supplementation confers little effect on the microbial population of the cecum of laying hens under the current experimental setup. Interestingly, a clear distinction between normal feed treated- and probiotic-treated groups (Beta diversity) was observed, demonstrating that *B. subtilis* can influence the microbial community in the cecum of laying hens. We observed an increased trend in the phyla *Firmicutes* in groups treated with *B. subtilis*, although no significant alteration was observed. Certain bacteria from *Firmicutes* may improve fermentation and further intestinal absorption through several mechanisms, and also affect short-chain fatty acid metabolism [37,38,39]. Currently, there is no general consensus on the effects of *B. subtilis* on the gut microbiota of laying hens at the phylum level. Cohering with our results, several studies have concluded that *B. subtilis* supplementation does not change the relative abundances of *Bacteroidetes* and *Firmicutes* in the ileal mucosa of laying hens [40,41]. Contrary to this, some researchers have demonstrated that *B. subtilis* intervention can effectively regulate the cecal microbiota’s composition [42,43]. For instance, Li et al. showed that, compared to a control group of broilers, *B. subtilis* CGMCC can increase the relative abundances of *Bacteroidetes* and *Proteobacteria*, and accordingly reduce the ratio of *Firmicutes* [42]. Several factors may contribute to the different conclusions drawn from these clinical trials described above, e.g., probiotic strain specificity, host background, probiotic administration period, or dose.

A further analysis of microbiota composition at the genus level showed that *B. subtilis* intervention can induce a health-promoting pattern in a series of genera of cecal microbiota. An increasing trend of *Lactobacillus* and a significant elevation of *Bifidobacterium* were observed in the *B. subtilis*-treated group, and these beneficial bacteria are believed to effectively modulate the gut microbiota and promote gut health [44,45,46]. An increase in beneficial bacteria in the gut could be attributed to the indirect action of *B. subtilis*, since *B. subtilis* can extensively consume oxygen in the gut and promote the growth of *Lactobacillus* and *Bifidobacterium* [47,48]. Similar results were reported in other studies. Liu et al. and Forte et al. found that *B. subtilis* supplementation can also increase the relative ratio of *Lactobacillus* in chicken’s guts [49,50]. Moreover, a considerable level of *Campylobacter* is present in chicken guts [51], and *Lactobacillus* can inhibit the colonization of *Campylobacter* in chicken’s digestive tracts [52]. We observed a lower level of *Campylobacter* in the *B. subtilis*-treated groups, although it is not clear whether there is a causative relationship between *Lactobacillus* and *Campylobacter*. In addition, we observed no significant increases in the relative abundance of *B. subtilis* in the *B. subtilis*-treated group. Generally, *Bacillus spp*. can only establish a transient colonization of the gastrointestinal tracts of hosts, differing from *Lactobacillus* [53,54,55]. We observed a significant reduction in the relative ratio of *Staphylococci* in the *B. subtilis*-treated groups, and similar results were observed in other trials [56]. *Staphylococci* infection can not only cause disease in chickens, but it also poses a threat to public health due to the presence of multiple antibiotic-resistant *Staphylococcus* strains [57,58].

Due to the extremely complex environment of the host’s gut, the mechanism by which *Bacillus spp.* inhibit pathogens in vivo remain obscure. Piewngam et al. demonstrated that the lipopeptide and fengycin derived from probiotic *Bacillus* can contribute to intestinal *S. aureus* decolonization by inhibiting the pathogen’s signaling pathway [59,60]. A significant reduction in the relative abundance of *Fusobacteria* was also observed in the *B. subtilis*-treated groups. Kollarcikova et al. discovered that the overgrowth of certain opportunistic bacteria, such as *Fusobacteria*, may be associated with the reduced performance of chickens, although a clear causal relationship has still yet to be established [61]. Currently, the exact effect of *Fusobacteria* on chicken health is still unclear, although we know it resides in the gut. Therefore, its contribution to gut homeostasis should be explored. In humans, some *Fusobacterium spp*. are implicated in a wide range of clinical diseases [62,63], and there is a risk of this pathogenic bacteria spreading from chickens to farmworkers.

The intestinal epithelial cells serve as a vital physical barrier against the external environment and stimuli, such as pathogens, toxin, etc. The integrity of the epithelium is maintained by a series of intercellular junctional complexes, consisting of tight junctions, adherens junctions, and desmosomes [64,65]. Occludin is an important tight junction protein, and it contributes to tight junction stabilization and optimal barrier functionality. *B. subtilis* significantly elevates occludin’s mRNA expression level in laying hens, suggesting it improves gut barrier function and optimizes the intestinal defense status. Similar results were also reported by other groups [29,66], showing the effects of *B. subtilis* on the intestinal tight junction protein. Several mechanisms may be implicated in *B. subtilis*’ regulation of epithelial cells’ expression of occludin. It is well known that probiotics can have inhibitory effects on gut pathogenic bacteria via adhesion with host cells [25]. Additionally, much evidence suggests that *B. subtilis* improves the expression of tight junction proteins by different mechanisms, including the mitigation of inflammatory cytokines expression (TNF-α, IL-6) by cyclic peptide secretion [67,68]. *Lactobacillus reuteri* can induce the expression of zonula occluden-1 and occludin through the myosin light-chain kinase pathway [69], but it is still not clear whether this pathway mediates *B. subtilis*’ regulatory effects on occludin. *B. subtilis* supplementation enhances epithelial barrier integrity not only in laying hens but also in broiler chickens, characterized by the increased expression of occludin at the mRNA or protein level in the gut [66,70].

TLRs, crucial pattern-recognition receptors in mammals and birds, play an important role in the innate immune response via engaging with the pathogen-associated molecular patterns of microorganisms, generally leading to the activation of genes related to anti- or pro-inflammatory cytokines and subsequent immune activities [71,72]. No statistically significant changes in the mRNA expression levels of TLR1, TLR2, TLR4, or TLR15 were observed in our current study, indicating that *B. subtilis* does not trigger TLR-mediated inflammatory activity. Alternatively, *B. subtilis* may be capable of reducing inflammation in the chicken gut. A further trial with bacterial challenge (such as *Salmonella* or *Campylobacter*) may elucidate the anti-inflammatory effects of *B. subtilis*. In agreement with our study, several groups demonstrated that dietary supplementation with probiotics (*Lactobacillus acidophilus*, *B. coagulans*) in chickens does not significantly affect the expression levels of TLRs in the gut [22,73]. In contrast, several groups have demonstrated that *B. subtilis* treatment induces an increase or reduction in the expression levels of these TLRs in broiler chickens or chicken dendritic cells [21,74,75,76]. The contradictory data described above may be attributed to several factors, such as experimental period, probiotic dose, probiotic composition, strain specificity, etc. Further exploration and refined studies are needed to clarify this issue.

In conclusion, the oral administration of higher amounts of probiotic *B. subtilis* (2.5 × 10^9^ CFU/kg) to laying hens for 4 weeks cannot improve overall egg production, can induce a healthy pattern in microbiota composition characterized by a higher level of beneficial bacteria, and induces a higher expression level of tight junction protein. Probiotic *B. subtilis* supplementation at lower levels (2.5 × 10^9^ CFU/kg) had fewer modulatory effects. Our study shows that the *B. subtilis* strain YW1 possesses health-promoting abilities, and can serve as a probiotic alternative to antibiotics in the poultry industry, as well as a reference strain for clinical application.

## Figures and Tables

**Table 1:** Composition of the experimental diets for laying hens (as-fed basis, %, unless otherwise indicated).

Ingredients		Nutrient Level ^2^	
Corn (%)	62.85	CP (%)	16.51
Soybean meal (%)	26.35	ME (MJ/kg)	11.16
Limestone (%)	9.00	Calcium (%)	3.4
CaHPO_4_ (%)	1.00	AP (%)	0.34
Premix ^1^ (%)	0.65	TP (%)	0.66
*DL*-Met (%)	0.15	Met	0.42
Total	100.00	Lys	0.86
		Met + Cys	0.72
		Lys/Met	2.06

^1^ The premix provided the following per kg to the diet: VA 12,500 IU, VD_3_ 4125 IU, VE 15 IU, VK 2 mg, thiamine 1 mg, riboflavin 8.5 mg, calcium pantothenate 50 mg, nicotinic acid 32.5 mg, pyridoxine 8 mg, VB_12_ 5 mg, biotin 2 mg, Fe (as ferrous sulfate) 60 mg, Cu (as copper sulfate) 8 mg, Zn (as zinc sulfate) 66 mg, Mn 65 mg, Se 0.3 mg, I 1 mg, choline 0.5 g, phytase 0.5 g, NaCl 3.0 g. ^2^ The data on nutrients depict the measured content except ME and non-phytate phosphorus.

**Table 2:** Sequence information of primers used for real-time PCR.

Genes	GenBank Number	Forward Sequence (5′ to 3′)	Reverse Sequence (5′ to 3′)	Reference
*β-Actin*	NM_205518.1	ACTCTGGTGATGGTGTTAC	GGCTGTGATCTCCTTCTG	[19]
*GAPDH*	AF047874	GAAGGCTGGGGCTCATCTG	CAGTTGGTGGTGCACGATG	[20]
*Occludin*	NM_205128.1	TCATCGCCTCCATCGTCTAC	TCTTACTGCGCGTCTTCTGG	[19]
*TLR1*	AB109401.1	GGCAGTGGACGCAGACAAA	GTAGGAAATGAAGGCGTGGAA	[21]
*TLR2*	NM_204.278	CTGGGAAGTGGATTGTGGA	AAGGCGAAAGTGCGAGAAA	[22]
*TLR4*	NM_001030693.1	TTCCAAGCACCAGATAGCAACATC	ACGGGTCACAGAAGAACTTAGGG	[23]
*TLR15*	JN112029.1	ATCCTTGTCGTTCTGGTGCTAA	TCAGTAGATGCTCCTTCGTCCA	[21]
*TNFSF15*	NM010245578	CCTGAGTATTCCAGCAACGCA	ATCCACCAGCTTGATGTCACTAAC	[22]
*LYZ*	NM 205.281	CTGGGAAACTGGGTGTGTGT	AGCGGCTGTTGATCTGTAGG	[22]

**Table 3:** Effects of probiotics on average egg production and weight over the whole experimental period (1~4 week).

Parameters	Ctr	T1	T2
Egg production (%), before treatment	92.64 ± 1.64	92.56 ± 3.35	93.60 ± 2.63
Egg production (%), after treatment	93.11 ± 1.44	95.99 ± 1.28	95.19 ± 1.58
Egg weight (g), before treatment	62.09 ± 0.72	62.71 ± 0.76	62.66 ± 1.03
Egg weight (g), after treatment	64.63 ± 0.96	63.99 ± 1.69	64.65 ± 0.50

Ctr: control group fed a basal diet; T1, T2: treatment groups fed a basal diet supplemented with *B. subtilis* at different doses (T1 = 5 × 10^8^ CFU/kg; T2 = 2.5 × 10^9^ CFU/kg). Data are shown as mean ± SD.

**Table 4:** Effects of *B. subtilis* supplementation on the morphology of the jejunum in laying hens at day 28 of the experiment.

Parameters	Ctr	T1	T2
Villus height (μm)	876.96 ± 232.38	869.42 ± 183.76	898 ± 248.37
Crypt depth (μm)	76.71 ± 26.73	76.02 ± 22.21	75.45 ± 21.05
Villus height/crypt depth ratio	11.43 ± 1.15	11.43 ± 3.91	11.93 ± 1.51

Ctr: control group fed a basal diet; T1: group fed a basal diet supplemented with *B. subtilis* (5 × 10^8^ CFU/kg); T2: group fed by basal diet supplemented with *B. subtilis* (2.5 × 10^9^ CFU/kg). Data are shown as mean ± SD.

## Data Availability

All data are available from the corresponding authors on reasonable request.
